# Supplementary material for: Acute febrile illness in Kenya: Clinical characteristics and pathogens detected among patients hospitalized with fever, 2017–2019
Source: PLoS One. 2024 Aug 1;19(8):e0305700. doi: 10.1371/journal.pone.0305700 (PMC11293630; doi:10.1371/journal.pone.0305700)

**S1 Figure. Map of surveillance sites.** Sites included Kenyatta National Hospital in Nairobi City County, Coast General Teaching and Referral Hospital in Mombasa County, Kakamega County Referral Hospital in Kakamega County, and Kakuma Refugee Camp General Hospital in Turkana County. Size of circle reflects bed capacity of the participating hospital in each site.


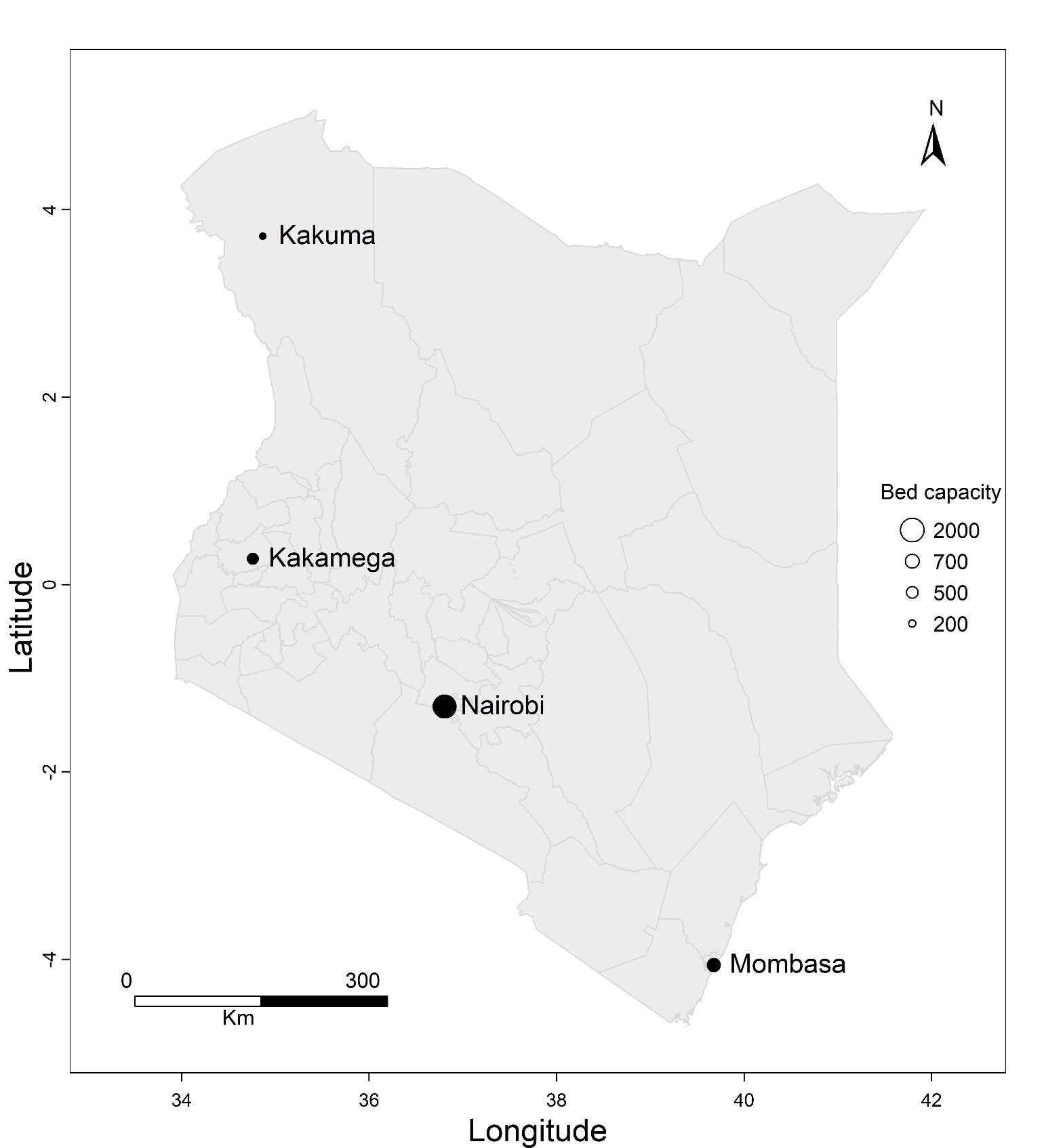

Supplement: S1 Fig — Sites included Kenyatta National Hospital in Nairobi City County, Coast General Teaching and Referral Hospital in Mombasa County, Kakamega County Referral Hospital in Kakamega County, and Kakuma Refugee Camp General Hospital in Turkana County. Size of circle reflects bed capacity of the participating hospital in each site. (DOCX) [file pone.0305700.s001.docx]
