# Supplementary figures and images for: Acute febrile illness in Kenya: Clinical characteristics and pathogens detected among patients hospitalized with fever, 2017–2019
Source: PLoS One. 2024 Aug 1;19(8):e0305700. doi: 10.1371/journal.pone.0305700 (PMC11293630; doi:10.1371/journal.pone.0305700)

**S2 Figure. TAC card targets**

**
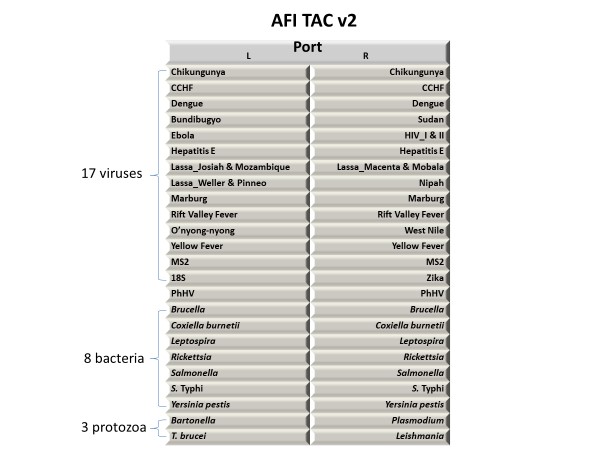
**

Supplement: S2 Fig — (DOCX) [file pone.0305700.s002.docx]
