# Supplementary material for: Acute febrile illness in Kenya: Clinical characteristics and pathogens detected among patients hospitalized with fever, 2017–2019
Source: PLoS One. 2024 Aug 1;19(8):e0305700. doi: 10.1371/journal.pone.0305700 (PMC11293630; doi:10.1371/journal.pone.0305700)
Supplement: S1 Table — (DOCX) [file pone.0305700.s003.docx]

**S1 Table 1 Discharge diagnoses among acute febrile illness (AFI) cases, at four public hospitals in Kenya, June 2017-March 2019^[[1]](#footnote-2)^**

|  | **Kakuma**  **(n=908)** | **Kakamega**  **(n=355)** | **Nairobi**  **(n=986)** | **Mombasa**  **(n=664)** | **Total**  **(n=2913)** |
| --- | --- | --- | --- | --- | --- |
| Pneumonia | 359 (39.5) | 87 (24.5) | 380 (38.5) | 239 (36.0) | 1065 (36.6) |
| Malaria | 254 (28.0) | 203 (57.2) | 65 (6.6) | 96 (14.5) | 618 (21.2) |
| Gastroenteritis | 165 (18.2) | 44 (12.4) | 180 (18.3) | 185 (27.9) | 574 (19.7) |
| Meningitis | 4 (0.4) | 33 (9.3) | 122 (12.4) | 83 (12.5) | 242 (8.3) |
| Febrile convulsions | 10 (1.1) | 9 (2.5) | 129 (13.1) | 63 (9.5) | 211 (7.2) |
| Neonatal sepsis | 0 | 4 (1.1) | 139 (14.1) | 7 (1.1) | 150 (5.1) |
| Acute febrile illness | 114 (12.6) | 1 (0.3) | 14 (1.4) | 14 (2.1) | 143 (4.9) |
| Anemia | 25 (2.8) | 36 (10.1) | 53 (5.4) | 56 (8.4) | 120 (4.1) |
| Severe acute malnutrition | 29 (3.2) | 0 | 58 (5.9) | 32 (4.8) | 119 (4.1) |
| Septicemia | 33 (3.6) | 11 (3.1) | 15 (1.5) | 48 (7.2) | 107 (3.7) |
| Rickets | 0 | 0 | 92 (9.3) | 7 (1.1) | 99 (3.4) |
| Sickle cell | 8 (0.9) | 21 (5.9) | 36 (3.7) | 13 (2.0) | 78 (2.7) |
| Malnutrition | 27 (3.0) | 4 (1.1) | 32 (3.2) | 10 (1.5) | 73 (2.5) |
| Pulmonary tuberculosis | 20 (2.2) | 6 (1.7) | 33 (3.3) | 12 (1.8) | 71 (2.4) |
| Bronchiolitis | 0 | 2 (0.6) | 27 (2.7) | 27 (4.1) | 56 (1.9) |
| Upper respiratory tract infection | 34 (3.7) | 1 (0.3) | 13 (1.3) | 6 (0.9) | 54 (1.9) |
| Kidney injury | 1 (0.1) | 0 | 48 (4.9) | 2 (0.3) | 51 (1.8) |
| Neonatal jaundice | 0 | 0 | 46 (4.7) | 2 (0.3) | 48 (1.6) |
| Heart disease | 1 (0.1) | 1 (0.3) | 21 (2.1) | 8 (1.2) | 31 (1.1) |
| Septic shock | 0 | 0 | 17 (1.7) | 3 (0.5) | 30 (1.0) |
| Cerebral palsy | 1 (0.1) | 3 (0.8) | 19 (1.9) | 7 (1.1) | 30 (1.0) |
| Urinary tract infection | 12 (1.3) | 4 (1.1) | 11 (1.1) | 1 (0.2) | 28 (1.0) |
| Meningoencephalitis | 0 | 0 | 19 (1.9) | 3 (0.5) | 22 (0.8) |
| HIV | 0 | 0 | 13 (1.3) | 4 (0.6) | 17 (0.6) |
| Asthma | 2 (0.2) | 1 (0.3) | 9 (0.9) | 1 (0.2) | 13 (0.4) |
| Liver disease | 1 (0.1) | 1 (0.3) | 5 (0.5) | 3 (0.5) | 10 (0.3) |
| Leptospirosis | 9 (1.0) | 0 | 1 (0.1) | 0 | 10 (0.3) |
| Dengue | 0 | 0 | 0 | 8 (1.2) | 8 (0.3) |
| Moderate acute malnutrition | 1 (0.1) | 0 | 0 | 5 (0.8) | 6 (0.2) |
| Amoebiasis | 4 (0.4) | 0 | 0 | 0 | 4 (0.1) |
| HIV exposed | 0 | 0 | 3 (0.3) | 1 (0.2) | 4 (0.1) |
| Viral encephalitis | 0 | 1 (0.3) | 0 | 0 | 1 (0) |
| Chikungunya | 0 | 0 | 0 | 1 (0.2) | 1 (0) |
| Others | 106 (11.7) | 39 (11.0) | 149 (15.1) | 54 (8.1) | 348 (12.0) |

1. Cases could have more than one discharge diagnosis [↑](#footnote-ref-2)
