# Supplementary material for: Acute febrile illness in Kenya: Clinical characteristics and pathogens detected among patients hospitalized with fever, 2017–2019
Source: PLoS One. 2024 Aug 1;19(8):e0305700. doi: 10.1371/journal.pone.0305700 (PMC11293630; doi:10.1371/journal.pone.0305700)
Supplement: S2 Table — (DOCX) [file pone.0305700.s004.docx]

**S2 Table 2. Pathogens detected by TAC among UF cases (n=1314) in Kenya at four public hospitals in Kenya, June 2017-March 2019**

| **TAC result** | **n** | **%** |
| --- | --- | --- |
| Negative | 715 | 54.41 |
| Plasmodium | 471 | 35.84 |
| Plasmodium+Chikungunya | 8 | 0.61 |
| Plasmodium+Bartonella | 1 | 0.08 |
| Plasmodium+Rickettsia | 5 | 0.38 |
| Plasmodium+HIV_1 | 11 | 0.84 |
| Plasmodium+Brucella | 1 | 0.08 |
| Plasmodium+Dengue | 5 | 0.38 |
| Plasmodium+Dengue+Rickettsia | 1 | 0.08 |
| Plasmodium+Leishmania | 1 | 0.08 |
| Plasmodium+Rift Valley Fever | 1 | 0.08 |
| Plasmodium+Salmonella | 1 | 0.08 |
| Plasmodium+Salmonella+Salmonella_Typhi | 1 | 0.08 |
| Plasmodium+Salmonella_Typhi | 2 | 0.15 |
| Rift Valley Fever | 1 | 0.08 |
| Salmonella Typhi | 5 | 0.38 |
| HIV_1 | 39 | 2.97 |
| HIV_1+Rickettsia | 1 | 0.08 |
| Rickettsia | 6 | 0.46 |
| Chikungunya | 22 | 1.67 |
| Dengue | 6 | 0.46 |
| Leishmania | 7 | 0.53 |
| Salmonella | 3 | 0.23 |
| **Total** | 1314 |  |
