# Supplementary material for: Acute febrile illness in Kenya: Clinical characteristics and pathogens detected among patients hospitalized with fever, 2017–2019
Source: PLoS One. 2024 Aug 1;19(8):e0305700. doi: 10.1371/journal.pone.0305700 (PMC11293630; doi:10.1371/journal.pone.0305700)
Supplement: S3 Table — (DOCX) [file pone.0305700.s005.docx]

**S3 Table 3. Pathogens detected by TAC among UF cases (n=1,314) by age group, June 2017-March 2019**

|  | Age group | | | | | Total |
| --- | --- | --- | --- | --- | --- | --- |
|  | <1 year (n=196) | 1-4 years (n=571) | 5-17 years (n=271) | 18-49 years (n=236) | 50+years (n=40) |  |
| Negative | 147 | 344 | 112 | 92 | 20 | 715 |
| Plasmodium | 40 | 184 | 126 | 107 | 14 | 471 |
| Plasmodium+Chikungunya | 2 | 4 | 2 | 0 | 0 | 8 |
| Plasmodium+Bartonella | 0 | 0 | 0 | 1 | 0 | 1 |
| Plasmodium+Rickettsia | 0 | 1 | 4 | 0 | 0 | 5 |
| Plasmodium+HIV_1 | 0 | 1 | 1 | 9 | 0 | 11 |
| Plasmodium+Brucella | 0 | 0 | 0 | 1 | 0 | 1 |
| Plasmodium+Dengue | 0 | 3 | 1 | 0 | 1 | 5 |
| Plasmodium+Dengue+Rickettsia | 0 | 0 | 1 | 0 | 0 | 1 |
| Plasmodium+Leishmania | 0 | 0 | 1 | 0 | 0 | 1 |
| Plasmodium+Rift Valley Fever | 0 | 0 | 0 | 1 | 0 | 1 |
| Plasmodium+Salmonella | 0 | 1 | 0 | 0 | 0 | 1 |
| Plasmodium+Salmonella+Salmonella_Typhi | 0 | 0 | 1 | 0 | 0 | 1 |
| Plasmodium+Salmonella_Typhi | 0 | 0 | 2 | 0 | 0 | 2 |
| Rift Valley Fever | 0 | 0 | 0 | 0 | 1 | 1 |
| Salmonella_Typhi | 1 | 2 | 1 | 1 | 0 | 5 |
| HIV_1 | 2 | 7 | 11 | 15 | 4 | 39 |
| HIV_1+Rickettsia | 0 | 0 | 0 | 1 | 0 | 1 |
| Rickettsia | 0 | 1 | 1 | 4 | 0 | 6 |
| Chikungunya | 3 | 15 | 3 | 1 | 0 | 22 |
| Dengue | 0 | 4 | 0 | 2 | 0 | 6 |
| Leishmania | 1 | 2 | 3 | 1 | 0 | 7 |
| Salmonella | 0 | 2 | 1 | 0 | 0 | 3 |
| **Total** |  |  |  |  |  | 1314 |
