# Supplementary material for: Acute febrile illness in Kenya: Clinical characteristics and pathogens detected among patients hospitalized with fever, 2017–2019
Source: PLoS One. 2024 Aug 1;19(8):e0305700. doi: 10.1371/journal.pone.0305700 (PMC11293630; doi:10.1371/journal.pone.0305700)
Supplement: S4 Table — (DOCX) [file pone.0305700.s006.docx]

**S4 Table 4. Pathogens detected by TAC among UF cases (n=1,314) by site, June 2017-March 2019**

| **TAC result** | **Study Site** | | | | **Total** |
| --- | --- | --- | --- | --- | --- |
|  | **Kakuma (n=515)** | **Kakamega (n=222)** | **Nairobi (n=311)** | **Mombasa (n=266)** |  |
| Negative | 260 | 94 | 248 | 113 | 715 |
| Plasmodium | 223 | 113 | 43 | 92 | 471 |
| Plasmodium+Chikungunya | 0 | 0 | 0 | 8 | 8 |
| Plasmodium+Bartonella | 1 | 0 | 0 | 0 | 1 |
| Plasmodium+Rickettsia | 2 | 1 | 2 | 0 | 5 |
| Plasmodium+HIV_1 | 3 | 1 | 1 | 6 | 11 |
| Plasmodium+Brucella | 1 | 0 | 0 | 0 | 1 |
| Plasmodium+Dengue | 0 | 0 | 0 | 5 | 5 |
| Plasmodium+Dengue+Rickettsia | 0 | 0 | 0 | 1 | 1 |
| Plasmodium+Leishmania | 1 | 0 | 0 | 0 | 1 |
| Plasmodium+Rift Valley Fever | 1 | 0 | 0 | 0 | 1 |
| Plasmodium+Salmonella | 1 | 0 | 0 | 0 | 1 |
| Plasmodium+Salmonella+Salmonella_Typhi | 0 | 0 | 1 | 0 | 1 |
| Plasmodium+Salmonella_Typhi | 0 | 2 | 0 | 0 | 2 |
| Rift Valley Fever | 0 | 0 | 0 | 1 | 1 |
| Salmonella_Typhi | 2 | 3 | 0 | 0 | 5 |
| HIV_1 | 8 | 6 | 13 | 12 | 39 |
| HIV_1+Rickettsia | 1 | 0 | 0 | 0 | 1 |
| Rickettsia | 4 | 1 | 0 | 1 | 6 |
| Chikungunya | 0 | 0 | 0 | 22 | 22 |
| Dengue | 0 | 1 | 2 | 3 | 6 |
| Leishmania | 7 | 0 | 0 | 0 | 7 |
| Salmonella | 0 | 0 | 1 | 2 | 3 |
| **Total** |  |  |  |  | 1314 |
