# Supplementary material for: Acute febrile illness in Kenya: Clinical characteristics and pathogens detected among patients hospitalized with fever, 2017–2019
Source: PLoS One. 2024 Aug 1;19(8):e0305700. doi: 10.1371/journal.pone.0305700 (PMC11293630; doi:10.1371/journal.pone.0305700)
Supplement: Attachment — Submitted filename: response to reviewer comments.docx [file pone.0305700.s007.docx]

May 7, 2024

Dear Dr. Roques,

Many thanks for sharing these reviewer comments. We have revised the manuscript to address their concerns and have prepared a point-by-point response to each comment below. We have also addressed the additional journal requirements noted below. Please let us know if anything else is needed. Thank you again for your consideration of this manuscript.

Regards,

Jennifer Verani

When submitting your revision, we need you to address these additional requirements.

1. Please ensure that your manuscript meets PLOS ONE's style requirements, including those for file naming. The PLOS ONE style templates can be found at
   <https://journals.plos.org/plosone/s/file?id=wjVg/PLOSOne_formatting_sample_main_body.pdf> and 
   <https://journals.plos.org/plosone/s/file?id=ba62/PLOSOne_formatting_sample_title_authors_affiliations.pdf>

**Author response:** We have revised the file names and the title page to adhere to PLOS ONE’s style requirements – apologies for having missed that with the initial submission.

2. We note that the grant information you provided in the ‘Funding Information’ and ‘Financial Disclosure’ sections do not match.

When you resubmit, please ensure that you provide the correct grant numbers for the awards you received for your study in the ‘Funding Information’ section.

**Author response:** Apologies for that inconsistency. We have aligned them in the resubmission.

3. When completing the data availability statement of the submission form, you indicated that you will make your data available on acceptance. We strongly recommend all authors decide on a data sharing plan before acceptance, as the process can be lengthy and hold up publication timelines. Please note that, though access restrictions are acceptable now, your entire data will need to be made freely accessible if your manuscript is accepted for publication. This policy applies to all data except where public deposition would breach compliance with the protocol approved by your research ethics board. If you are unable to adhere to our open data policy, please kindly revise your statement to explain your reasoning and we will seek the editor's input on an exemption. Please be assured that, once you have provided your new statement, the assessment of your exemption will not hold up the peer review process.

**Author response:** At the time of submission, we anticipated being able to make the data publicly available without restrictions. However, the Data Protection Act ([TheDataProtectionAct__No24of2019.pdf (kenyalaw.org)](https://kenyalaw.org/kl/fileadmin/pdfdownloads/Acts/2019/TheDataProtectionAct__No24of2019.pdf)), which was enacted in Kenya in 2019 and more strictly enforced in recent years, states that:

53. Research, history and statistics (1) The further processing of personal data shall be compatible with the purpose of collection if the data is used for historical, statistical or research purposes and the data controller or data processor shall ensure that the further processing is carried out solely for such purposes and will not be published in an identifiable form. (2) The data controller or data processor shall take measures to establish appropriate safeguards against the records being used for any other purposes.

The data presented in this manuscript have several stewards. The Kenya Medical Research Council (KEMRI) provided the primary ethical approval for the work (via the KEMRI Scientific and Ethical Review Unit) and owns the data. Washington State University was the implementing partner at the time of data collection and was responsible for safekeeping and management of the data. The Kenya Ministry of Health has been involved in the surveillance from its inception and is currently assuming leadership of the platform; therefore, moving forward the data will be co-owned by the Ministry of Health and KEMRI. After discussion among co-authors representing the various institutions and considering the Data Protection Act, we unfortunately cannot make the data publicly without any assurance that the data will be used in accordance with the aims of the protocol under which they were collected. The data can be made available through a process of managed access requiring the submission of a request for consideration by the KEMRI Data Governance Committee via email cghr@kemri.go.ke or telephone +254 (20) 22923.

We will revise our statement on data availability and request that you please seek input form an editor on a possible exception to this policy.

4. We note you have included a table to which you do not refer in the text of your manuscript. Please ensure that you refer to Table 2 in your text; if accepted, production will need this reference to link the reader to the Table.

**Author response:** We have added a call-out to Table 2 in the relevant paragraph of the Results section.

5. We note that [Figure S1] in your submission contain [map/satellite] images which may be copyrighted. All PLOS content is published under the Creative Commons Attribution License (CC BY 4.0), which means that the manuscript, images, and Supporting Information files will be freely available online, and any third party is permitted to access, download, copy, distribute, and use these materials in any way, even commercially, with proper attribution. For these reasons, we cannot publish previously copyrighted maps or satellite images created using proprietary data, such as Google software (Google Maps, Street View, and Earth). For more information, see our copyright guidelines: <http://journals.plos.org/plosone/s/licenses-and-copyright>.
 
We require you to either (1) present written permission from the copyright holder to publish these figures specifically under the CC BY 4.0 license, or (2) remove the figures from your submission:

**Author response:** Thank you for noting this issue with the copyrighted image. We have generated another map using R software that should not have copyright issues.
 
a. You may seek permission from the original copyright holder of Figure S1 to publish the content specifically under the CC BY 4.0 license.  
 
We recommend that you contact the original copyright holder with the Content Permission Form (<http://journals.plos.org/plosone/s/file?id=7c09/content-permission-form.pdf>) and the following text:
“I request permission for the open-access journal PLOS ONE to publish XXX under the Creative Commons Attribution License (CCAL) CC BY 4.0 (<http://creativecommons.org/licenses/by/4.0/>). Please be aware that this license allows unrestricted use and distribution, even commercially, by third parties. Please reply and provide explicit written permission to publish XXX under a CC BY license and complete the attached form.”
 
Please upload the completed Content Permission Form or other proof of granted permissions as an "Other" file with your submission.
 
In the figure caption of the copyrighted figure, please include the following text: “Reprinted from [ref] under a CC BY license, with permission from [name of publisher], original copyright [original copyright year].”
 
b. If you are unable to obtain permission from the original copyright holder to publish these figures under the CC BY 4.0 license or if the copyright holder’s requirements are incompatible with the CC BY 4.0 license, please either i) remove the figure or ii) supply a replacement figure that complies with the CC BY 4.0 license. Please check copyright information on all replacement figures and update the figure caption with source information. If applicable, please specify in the figure caption text when a figure is similar but not identical to the original image and is therefore for illustrative purposes only.
The following resources for replacing copyrighted map figures may be helpful:
 
USGS National Map Viewer (public domain): <http://viewer.nationalmap.gov/viewer/>
The Gateway to Astronaut Photography of Earth (public domain): <http://eol.jsc.nasa.gov/sseop/clickmap/>
Maps at the CIA (public domain): <https://www.cia.gov/library/publications/the-world-factbook/index.html> and <https://www.cia.gov/library/publications/cia-maps-publications/index.html>
NASA Earth Observatory (public domain): <http://earthobservatory.nasa.gov/>
Landsat: <http://landsat.visibleearth.nasa.gov/>
USGS EROS (Earth Resources Observatory and Science (EROS) Center) (public domain): [http://eros.usgs.gov/#](http://eros.usgs.gov/)
Natural Earth (public domain): <http://www.naturalearthdata.com/>

6. Please include captions for your Supporting Information files at the end of your manuscript, and update any in-text citations to match accordingly. Please see our Supporting Information guidelines for more information: <http://journals.plos.org/plosone/s/supporting-information>.

**Author response:** We have added captions for the Supporting Information after the references in the manuscript, and have updated the in-text citations.

Reviewers' comments:

Reviewer's Responses to Questions

**Comments to the Author**

1. Is the manuscript technically sound, and do the data support the conclusions?

The manuscript must describe a technically sound piece of scientific research with data that supports the conclusions. Experiments must have been conducted rigorously, with appropriate controls, replication, and sample sizes. The conclusions must be drawn appropriately based on the data presented.

Reviewer #1: Yes

2. Has the statistical analysis been performed appropriately and rigorously?

Reviewer #1: Yes

3. Have the authors made all data underlying the findings in their manuscript fully available?

The [PLOS Data policy](http://www.plosone.org/static/policies.action#sharing) requires authors to make all data underlying the findings described in their manuscript fully available without restriction, with rare exception (please refer to the Data Availability Statement in the manuscript PDF file). The data should be provided as part of the manuscript or its supporting information, or deposited to a public repository. For example, in addition to summary statistics, the data points behind means, medians and variance measures should be available. If there are restrictions on publicly sharing data—e.g. participant privacy or use of data from a third party—those must be specified.

Reviewer #1: No

4. Is the manuscript presented in an intelligible fashion and written in standard English?

PLOS ONE does not copyedit accepted manuscripts, so the language in submitted articles must be clear, correct, and unambiguous. Any typographical or grammatical errors should be corrected at revision, so please note any specific errors here.

Reviewer #1: Yes

5. Review Comments to the Author

Please use the space provided to explain your answers to the questions above. You may also include additional comments for the author, including concerns about dual publication, research ethics, or publication ethics. (Please upload your review as an attachment if it exceeds 20,000 characters)

Reviewer #1: General Comments

This is an expansive and interesting study that delves into the pathogens associated with acute febrile illness, and particularly “undifferentiated fever” (i.e. excluding diarrheal disease and/or lower respiratory disease) across four different geographical settings in Kenya. It is limited by the number of pathogens tested for and the fact that only blood specimens were collected and tested. Further depth could also have been undertaken with respect to analysing risk factors, i.e. for more common infections (malaria) as well as perhaps by grouping similar pathogens. However, the limitations are in fact an important component, and do a good job of highlighting the challenges of conducting comprehensive AFI surveillance – there are just too many pathogens that are associated with fever, and too many biological specimens that would need to be collected and tested to look for them all! There are enormous opportunities in standardizing methodologies and approaches for AFI surveillance and characterization, and this paper adds nicely to that literature.

**Author response:** We thank the reviewer for their comments.

Specific Comments

- Lines 74-78: When a patient is “admitted”, does this refer to both in-patient and out-patient care? The implication from the language is in-patient only, in which case it would be helpful to describe why individuals presenting with fever who were deemed eligible for out-patient care were not considered eligible. Or is this because eligibility was determined by review of logs, and so most out-patients would have already left the hospital by the time the surveillance staff tried to locate them? This could be worth clarifying, as it would help to explain the quite high percentage of patients that the surveillance staff were not able to locate. It could also have implications for some of the findings presented later, particularly with respect to mortality.

**Author response:** This study was restricted to hospitalized in-patients only. Although we initially considered enrolling both in-patients and out-patients, we did not have adequate resources to manage the logistics of comprehensive screening of all potentially eligible in-patients and out-patients. We chose to focus our efforts on the more severe manifestations of AFI, i.e. among persons requiring admission. The eligible hospitalized patients that could not be located were primarily patients that were discharged or transferred to another facility before the field team was able to enroll them.

- Line 131: Typically, “assent” refers to verbal agreement; do the authors mean to say that verbal assent was documented in writing? Or should this be changed to “written consent” being obtained?

**Author response:** Assent refers to a willingness to participate in research given by persons too young to legally give informed consent but old enough to understand the proposed research, study procedures and potential risks and benefits. We obtained written assent (in addition to written consent from parent or guardian) for participants aged 7 to 17 years.

- Line 140: Was the lack of blood collection in 30% of cases due to refusal by the patient or for other reasons?

**Author response:** Failure to obtain blood for culture was primarily due to either refusal or inability to obtain specimen; the latter was particularly a challenge in young children.

- Line 140-150: Were there any cases that were excluded due to signs of obvious injury as the cause of the infection (often an exclusion factor in AFI studies – see for example <https://www.ncbi.nlm.nih.gov/pmc/articles/PMC9436081/>), or other routine childhood infections not typically associated with transmissible pathogens (i.e. otitis)? The authors do note that a number of common and endemic viral causes of fever (i.e. enteroviruses, adenoviruses) were not considered here which is definitely a limitation, but there might be non-infectious causes that should be considered as well.

**Author response:** We excluded patients for whom the primary reason for seeking care was injury or trauma. We have edited the methods section to reflect this exclusion criterion. We did not exclude children with acute otitis media (which is generally caused by transmissible pathogens, e.g. *Streptococcus pneumoniae*, *Haemophilus influenzae* and *Moraxella catarrhalis*). During the pilot phase of the surveillance, we did originally have an additional criterion for undifferentiated fever (UF) cases (i.e. those who had blood samples collected) that was “absence of a clear focus of fever based on history and physical examination” (which might have included acute otitis media) so that our etiologic testing would focus on patients without a clear cause for their fever; however we found this criterion very difficult to operationalize consistently and therefore collected blood from eligible UF cases even if infections such as acute otitis media were manifest. While it is possible that there might have been some non-infectious causes of fever (e.g. oncologic or rheumatologic diseases) among patients meeting the study AFI criteria, the vast majority of those enrolled had a discharge diagnosis for one or more infectious illnesses.

- Lines 161-170: I appreciate that most individual diseases were detected too infrequently for robust risk factor analysis, but presumably this could have been done for malaria? Likewise the authors could have considered grouping similar pathogens (i.e. arboviruses) to look for risk factors associated with pathogens with similar ecological drivers and transmission patterns.

**Author response:** Risk factor analyses were not a focus of this primary analysis of the data, but may be included in a subsequent manuscript focused on malaria; for other pathogens (or even groups of pathogens), the power of such analyses would be very limited. Furthermore, because the available comparison group (persons without specific pathogens detected) had febrile illness due to some other cause, such case-case comparisons can yield associations that are difficult to interpret.

- Line 195-196: Suggest highlighting Figure 4 and seasonal patterns more clearly in the results section, it’s a bit hidden here but is potentially important. I appreciate that the authors consider the data to be too sparse for specific pathogens to conduct seasonal analyses (see Line 281) but there are additional qualitative observations that could be made; also there are sufficient data on overall number of eligible patients over time that could potentially be used to look for seasonal trends, segregated by region, in number of AFI cases presenting to the hospitals.

**Author response:** We appreciate the reviewer’s interest in the potential for detecting seasonal patterns in or data. However, since we had data from all four sites for only 15 months, we are reluctant to draw inferences about seasonal differences in overall case counts. Subsequent analyses of the data from the platform will provide richer insight into seasonality. As highlighted in the text of the results, we felt that the most notable temporal finding was the detection of Chikungunya cases after enrollment began in Mombasa, with a tapering off of those cases by the end of 2018.

- Line 227-229: This is an important point and one that perhaps could be highlighted further, particularly since quite a number of other studies focused on undifferentiated fever in malaria-endemic settings will exclude patients with a positive malaria RDT – despite the fact that sub-clinical malaria may abound and thus Plasmodium infection actually isn’t contributing to symptoms. Researchers working on AFI should be encouraged to include malaria-positive patients in their studies to ascertain if there might be co-infections causing AFI.

**Author response:** We agree with the reviewer about the importance of this point, and have added the following sentence to the end of that paragraph: “While many AFI studies exclude patients with malaria to focus on non-malarial etiologies of fever, our study highlights the importance of capturing data on malaria, including co-detection of malaria together with other pathogens, as well as the complexities of interpreting Plasmodium positivity across diverse etiologic contexts.”

- Line 274: Blood culture can also potentially can be biased by prophylactic antibiotic usage; was treatment history or reported self-medication included on the intake questionnaire?

**Author response:** We have added self-reported antibiotic use in the prior 7 days to Table 1, and a sentence about the frequency of prior antibiotic use to the third paragraph of the results section. We also added wording to the fifth paragraph of the discussion about antibiotics negatively impacting the sensitivity of blood culture.

- Table 1: How was the questionnaire developed and validated? For animal exposure, contact with rodents (i.e. presence of rodents in the house; recent sightings of rodents) would seem to be an important variable to include; likewise perhaps having a separate category for other wildlife. How was “contact” with animals defined?

**Author response:** The questionnaire was pilot tested and revised before launching the surveillance, but it was not formally validated. The question about animals was phrased as “Has the participant had contact with these animals during the last 2 weeks?” followed by a list of common animals (e.g. cow, goat, sheep, poultry) and space to capture contact with other animals. We did not, unfortunately, ask specifically about contact with rodents, but will consider adding that moving forward.

- Figure 2: This is a helpful chart to highlight the large proportion of cases in which no pathogen was detected, but not very helpful for visualizing the breakdown of identified pathogens/co-infections. Consider re-doing the chart to show only cases in which one or more pathogens were detected, to spread out the colors more evenly and allow readers better granularity of information. Figure 3 can remain as is to highlight the large proportion of cases with no pathogen detected (which is more interesting to see split across age groups, in any case).

**Author response:** We appreciate the reviewer’s feedback about the visualization of the identified pathogens. However, we feel that the high proportion of cases with no pathogen detected is important to highlight visually, and therefore prefer to maintain the current format of Figure 2. The details that the reviewer is requesting on the frequency of detection of individual pathogens and co-detection of pathogens are more easily found in the supplemental tables (which it seems the reviewer could not access).

- Supplemental tables: The supplemental materials do not seem to be available for review which is a shame, as I wanted to see what additional information was provided, especially Tables S1 and S2. In addition, the authors note assistance from the journal would be required to make underlying data available – one option would be to submit the manuscript, including underlying de-identified databases, to a pre-print server (i.e. <https://www.medrxiv.org/>) or online repository (i.e. <https://osf.io/>; also suitable for pre-prints).

**Author response:** The supplemental material was submitted along with the manuscript and main tables and figures. We are sorry that the review was not able to access them (and apologize if we inadvertently did something to make the inaccessible). We appreciate the reviewer’s suggestion about how to make the data publicly available.

6. PLOS authors have the option to publish the peer review history of their article ([what does this mean?](https://journals.plos.org/plosone/s/editorial-and-peer-review-process#loc-peer-review-history)). If published, this will include your full peer review and any attached files.


If you choose “no”, your identity will remain anonymous but your review may still be made public.


**Do you want your identity to be public for this peer review?** For information about this choice, including consent withdrawal, please see our [Privacy Policy](https://www.plos.org/privacy-policy).

Reviewer #1: **Yes:** Claire Standley
